# Transabdominal migration of a gossypiboma to the thoracic cavity: A case report

**DOI:** 10.1016/j.ijscr.2024.110486

**Published:** 2024-10-19

**Authors:** Mohammad Al-Jawad, Zein Almasri, Joud akroum, Hilal matar, Hasan dawod, Abdulmonem kawas

**Affiliations:** University of Aleppo, Faculty of Medicine, Aleppo, Syria

**Keywords:** Gossypiboma, Textiloma, Thoracic cavity, Liver surgery, Case report

## Abstract

**Introduction:**

Textiloma, or gossypiboma, is a rare oversight in thoracic surgery involving retained surgical sponges, with the potential for serious complications like chronic pain and infection. This case highlights a 29-year-old woman with a transdiaphragmatic migrated gossypiboma following liver surgery.

**Case presentation:**

A-29 years old woman presented with dyspnea, abdominal pain, and fever, with imaging revealing a suspected hepatic abscess. Surgical intervention identified a 15 cm gossypiboma in the thoracic cavity, previously misdiagnosed, leading to successful extraction and recovery.

**Discussion:**

Gossypibomas can cause significant complications, including abscess formation and respiratory distress, especially when migrating through the diaphragm. Diagnosis is challenging, often requiring advanced imaging techniques to identify the retained foreign body.

**Conclusion:**

This case emphasizes the need for heightened awareness of gossypiboma in patients with unexplained post-surgical symptoms. Early detection and meticulous surgical practices are crucial to prevent serious complications associated with retained foreign bodies.

## Highlights

• This case reports a gossypiboma migrating from the abdomen to the thorax post-liver surgery.
• The patient had respiratory distress and abdominal pain, revealing a retained surgical sponge.
• This underscores the need for awareness and meticulous surgical practices to avoid complications.

## 1 Introduction

Textiloma, also known as gossypiboma, refers to the oversight of a compress at the surgical site and is uncommon in thoracic surgery. Even more unusual is the transdiaphragmatic migration of a foreign body following abdominal surgery. Intrathoracic textiloma represents a rare yet serious consequence of surgical negligence, potentially leading to significant medical complications such as chronic pain, infection, and abscess formation. [1,2]

It is estimated to occur in approximately 1 in 1000 to 1 in 10,000 surgeries, [3] it can be diagnosed at varying intervals following surgery, with longer retention times associated with increased morbidity. [4]

It is important to highlight that in our case, we present a 29-year-old adult female with textiloma in the chest after surgery on the liver a 10-years ago, and we highlight the challenges encountered in managing this unique case as per the SCARE checklist. [5]

## 2 Case presentation

A 29-year-old woman, married with two children, presented to our hospital with complaints of dyspnea, right upper quadrant abdominal pain, right-sided chest pain, and fever. She reported weight loss, decreased appetite, and dysuria for the past month. Her surgical history includes the removal of a liver cyst ten years ago, and she has no significant past medical history and is not on any medications.

**Fig. 1:**
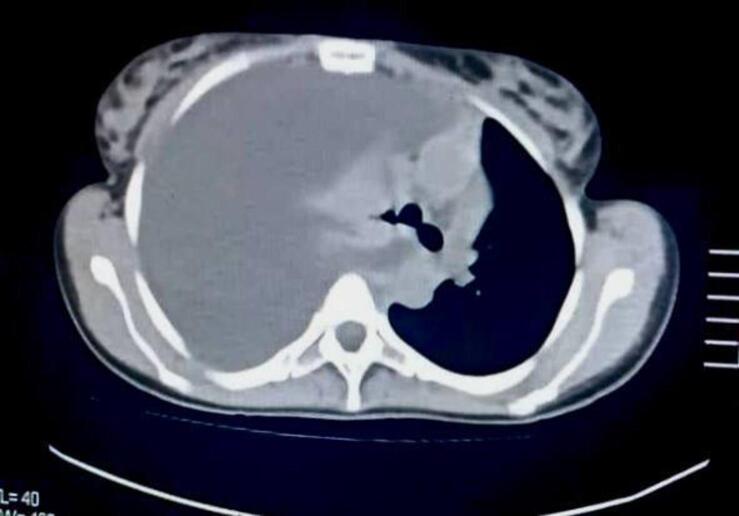
computed tomography (CT) which indicated a cavity likely containing a foreign body.

On examination, her pulse was 120–130 beats per minute, and her oxygen saturation was 97 % and her body temperature was 38.5 °C. Ultrasound examination revealed the presence of a hepatic abscess, followed by abdominal computed tomography (CT) which indicated a cavity likely containing a foreign body **(**Fig. 1**)**. Consequently, the patient was referred to the general surgery department for exploratory laparotomy.

A complete blood count (CBC) revealed slightly decreased lymphocyte levels at 0.43 × 10^9/L, while white blood cell, platelet, and hemoglobin levels were within normal limits. The chemistry panel indicated mildly decreased sodium levels at 132 mEq/L, with potassium levels remaining normal.

**Fig. 2:**
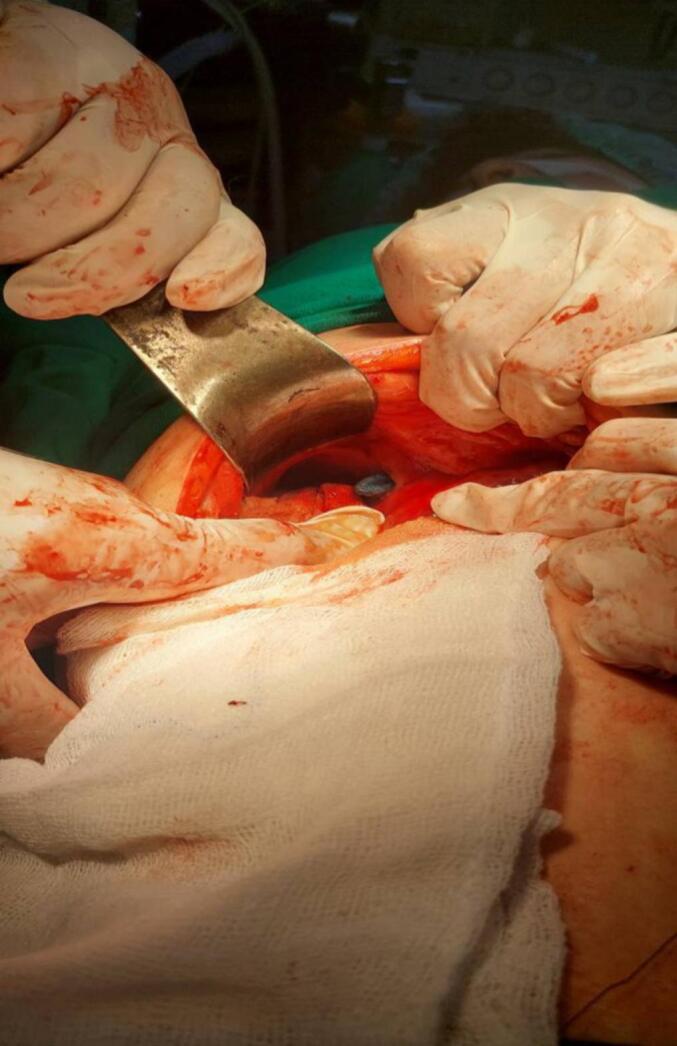
The image depicts the surgical site following open surgery to remove a foreign body identified as a gossypiboma,

The patient underwent open surgery to remove the foreign body. It was identified as a gossypiboma, previously misdiagnosed as a hepatic abscess **(**Fig. 2**)**. The gossypiboma was a retained surgical sponge from the prior liver cyst removal that had migrated to the thoracic cavity, measuring 15 cm × 11 cm. It was located in the right hemithorax, adjacent to the cardiac silhouette, exerting pressure on the right atrium, with associated right pleural effusion causing dilation of the inferior vena cava and hepatic veins.

An incision was made to access the abdominal cavity, where severe adhesions were encountered and released. The liver was mobilized away from the diaphragm, and a puncture of the diaphragm revealed purulent fluid. Upon opening the pleura, over 3 I of purulent fluid were evacuated, and the gossypiboma was located within the chest cavity. It was extracted, and the chest cavity was thoroughly irrigated. A chest tube was placed below the lung hilum, the diaphragm was closed with 2–0 nylon sutures, and a round drain was placed in Morrison's pouch.

Postoperatively, the CBC showed an increase in white blood cell count to 23 × 10^9/L. The patient was started on intravenous antibiotics, specifically Cefotazidime at a dosage of 2 g every 8 h and her condition improved, leading to her discharge from the hospital after 1 week without any complications.

## 3 Discussion

extiloma, or gossypiboma, is defined as a retained surgical sponge within the surgical bed. Surgical sponges are typically made of inert cotton; however, they can provoke an aseptic reaction, leading to the formation of foreign-body granulomas, fibrosis, adhesions, calcification, and ulceration. This condition is often asymptomatic and may be discovered incidentally. In some instances, the sponges can become infected, resulting in abscess formation and fistulization to adjacent structures. [2]

Gossypibomas are most commonly reported following laparotomy, but they can arise as complications of nearly any surgical procedure. Pathologically, they may present as either an aseptic fibrous response, leading to adhesions, encapsulation, and granuloma formation, or as an exudative reaction resulting in abscess formation. It is relatively rare to encounter an iatrogenic foreign body following thoracic surgery. Furthermore, the migration of a foreign body through the diaphragm after abdominal surgery, particularly after hepatic procedures, is exceptional. The contact between the foreign body and the diaphragm, facilitated by the pressure gradient between the thoracic and abdominal cavities, can lead to ischemia of the diaphragm muscle fibers and their erosion, ultimately resulting in the fistulization of the sponge into the thoracic cavity. [6]

In cases of thoracic textiloma, the clinical presentation is typically characterized by fever, purulent sputum, chest pain, and hemoptysis, often resulting from a bronchial fistula. [7]

Diagnosing a retained or migrated surgical sponge poses challenges due to the fibrotic foreign body reaction surrounding the gauze and the absence of distinct characteristics. Plain radiographs often fail to identify the sponge because of the lack of a radio-opaque marker. The hallmark of diagnosing a surgical sponge on a CT scan is a well-circumscribed lesion with a densely enhancing wall and a central low-density area that appears whirl-like, due to gas trapped within the fiber meshwork of the gossypiboma. On ultrasonography, the diagnostic feature is an intense and sharply defined acoustic shadow, which may be present even in the absence of air or calcification. [8]

## 4 Conclusion

This case underscores the importance of considering gossypiboma as a potential diagnosis in patients presenting with unexplained symptoms following surgical procedures. The migration of a retained surgical sponge can lead to serious complications such as abscess formation and respiratory distress. Clinicians should be vigilant about the possibility of retained foreign bodies, as early recognition and intervention can prevent further complications. This case serves as a reminder of the importance of meticulous surgical practices, including accurate counts of sponges and instruments.

## Authors contribution

The work's conception and design: all authors.

paper writing, and article revision: all authors.

Final revision and approval: all authors.

## Informed consent

Written informed consent was obtained from the patient for publication and any accompanying images.

## Consent for publication

All authors provide consent for publication.

## Sources of funding

There are no funding sources.

## Ethical approval

This case report does not require ethical approval as it involves a single patient case that is anonymized and does not include any identifiable personal information. The patient provided informed consent for the publication of this report.

## Provenance and peer review

Not commissioned, externally peer-reviewed.

## Registration of research studies

Our research study does not involve human subjects.

## Guarantor

Abdulmonem kawas

## Conflicts of interest

The authors declare that they have no competing interests.
